# Social determinants of inequities in under-nutrition (weight-for-age) among under-5 children: a cross sectional study in Gumla district of Jharkhand, India

**DOI:** 10.1186/s12939-016-0392-y

**Published:** 2016-07-08

**Authors:** Keya Chatterjee, Rajesh Kumar Sinha, Alok Kumar Kundu, Dhananjay Shankar, Rajkumar Gope, Nirmala Nair, Prasanta K Tripathy

**Affiliations:** Ekjut- NGO in India, Chakradharpur, West Singhbhum, Jharkhand India

**Keywords:** Underweight, Social determinants, Inequity, Tribal, Gender, Poverty, Maternal education

## Abstract

**Background:**

Jharkhand, a state with substantial tribal population in Eastern India has very high rate of undernutrition. The study tries to understand the social determinants of inequities in under-nutrition (weight-for-age) among children aged less than 5 years, in Gumla District of the State.

**Methods:**

Cross sectional study of 1070 children from 32 villages of 4 Blocks of Gumla District.

**Results:**

54.3 % (95 % CI 51.3–57.3) children were found to be underweight (less than -2SD), with insignificant difference between girls and boys. Multivariate analysis showed that poverty was the single most important predictor of undernutrition, where a child from the poorest quintile was 70 % more likely to be underweight (aOR 1.70, CI 1.13–2.57), compared to one from the least poor group (Quintile 5). While the difference in weight-for-age status between Scheduled Tribes and “OBC and other communities” was non-significant (95 % OR 1.12, CI 0.88–1.42) in the study context; community disaggregated data revealed that there were large variations within the tribal community, and numerically smaller communities also ranked lower in wealth, and their children showed poorer nutritional status. Other factors like maternal education beyond matriculation level also had some bearing. Bivariate analysis showed that chances of a child being underweight (<−2SD) was 43 % more and being severely underweight (<−3SD) was 26 % more for mothers with less than 10 years of schooling compared to those who had attended school for more than 10 years. Educational attainment of mothers did not show any significant difference between tribal and non-tribal communities.

**Conclusion:**

Overall nutritional status of children in Gumla is very grim and calls for immediate interventions, with universal coverage. Risk was almost equal for both genders, and for tribal and non-tribal population, though within tribal communities, it was slightly higher for smaller tribal communities, calling for soft targeting. Comprehensive programme addressing poverty and higher education for girls would be important to overcome the structural barriers, and should be integral part of any intervention. The study highlights the importance of soft targeting vulnerable communities within the universal coverage of government programmes for better nutritional outcomes.

## Background

The first 5 years of life play a critical role in defining a child’s physical and cognitive development that has an impact on the potential attainments in adult life. Good health and nutritional status, stimulating home environment of the child is very important in these formative years. The disadvantaged children are more likely to drop out of school, have reduced economic opportunity that spirals into intergenerational transmission of poverty [1].

Under nutrition is a result of interplay between different proximate, underlying and basic causes. While the immediate causes are inadequate dietary intake and diseases that operate at individual level, these factors are determined by underlying causes such as household food insecurity, poor living conditions, inadequate care of mothers and children and low access to health services. Factors embedded in the socio-economic-political context and structures determine how the downstream factors play out to differentially for communities and individuals, causing health inequities [2, 3]. WHO has defined social determinants of health inequities as “the interplay of socio-economic political context generating social stratifications and resulting socioeconomic position of individuals.” Within the social structure there are stratifiers that influence how the downstream intermediary determinants will play out for individuals or communities. It has identified income, occupation, education, social class, gender and race/ ethnicity as most important social stratifiers [4]. This paper tries to examine social determinants causing inequities in nutritional outcomes among children in Gumla. Weight for age was taken as a composite index of both weight for height and height for age, as a measure of nutritional status [5].

Gumla is located about 100 Kms from Ranchi, the capital city of Jharkhand. It is covered with dense forests and hills. About 95 % of the population lives in rural areas. The district has a population of 1.02 million, and a total literacy rate of 66.9 %.^1^ About 60 % of the population in this district come from economically weaker sections.^2^ Major occupations are agriculture, daily wage work, mining and forest produce collection. The State has been facing civil strife since the last decade.

Gumla has predominantly tribal population (68 %) while the remaining population constitutes of people from Other Backward Classes (OBC) and a very small proportion of General Castes. The tribal community in Gumla is further divided into different tribal groups, with Oraon (47 %), Munda (21 %) and Khariya (16 %) being among the larger tribal groups and the smaller groups are Baraik, Chik Baraik, Asur, Korva, Birijia (the last three being categorised as Particularly Vulnerable Tribal Groups (PVTGs). The study categorised social groups as ‘OBC and General’, ‘Oraons’, ‘Munda’, ‘Khariya’ and numerically smaller ‘other Scheduled Tribe groups’. PVTG population is sparsely present in two of the sampled Blocks and could not be included in the study due to their very small numbers; a separate study will be needed to cover this ultra-marginalised group. The study examined if there was a difference in weight-for-age by ethnicity (i.e., between tribal and non-tribal and between different tribal sub-groups of the population).

Economic condition of a household influences its access to quality living condition and services needed to maintain good health. It affects material resources at disposal of the household, also influencing its esteem and social standing relevant for participation in society [6]. Income and occupation could be a good indicators of economic condition, yet sometime it is difficult to obtain reliable information, when a large section of population is engaged in informal sector; in such cases proxy measures like living standards are used [7]. Principal Component Analysis is increasingly being used to differentiate socio-economic status (SES) within a population [8, 9].

Maternal education has shown to be a significant predictor of child survival and nutritional status [10, 11]. Higher maternal education could increase her control over household resources and health choices for her children; she can use simple health knowledge and better negotiate with the environment and health systems [12]. Caregiver’s knowledge and beliefs, control over resources and autonomy, workload and social support have a direct bearing on the health status of children [13]. For instance, immunisation status of children has been higher where mothers were educated [14–16]. The study has tried to explore if there is any influence of maternal education on child’s weight-for-age status.

## Methods

This cross sectional study covering 1070 children aged 6–59 months was undertaken between October and November 2014 to determine the prevalence of under-weight among children. Survey was conducted in 32 villages from 4 community development blocks- Raidih, Chainpur, Kamdara and Basia, of the District.

### Sampling strategy

Sample size calculation was made using prevalence of underweight of 63 % in rural Jharkhand (NFHS-3). Precision taken at 7.5 %, design effect 1.5 and individual response rate of 90 %. Sample per Block (strata) is 267. Hence, for 4 strata, sample size was calculated at 1070 for children under 5 years of age.

3 stage sampling process was followed. In the first stage, purposive sampling was done to identify 4 Blocks of Gumla district. In the second stage, 8 villages from each Block (Strata) were selected using Population Proportion to Size (PPS) method. From each of these villages, names of children were collected from Anganwadi centres and also by snowballing with members of women’s groups, ASHAs (Accredited Social Health Activist) and ANMs (Auxilliary Nurse Midwives) to identify any child who may have been left out from the Anganwadi list.

In the third stage, 33–34 households from each village with children of the target age group were selected using computer generated random numbers. 194 respondents had to be replaced as they were not available or had migrated. New respondents were identified using random sampling without replacement method. There were no cases of refusal to participate in the survey.

### Data collection and analysis

A simple questionnaire with close ended dichotomous or multiple-choice questions, covering identification of the child, age, weight, social group, parental educational status, household assets and peer group association was administered. Data collection was done by trained investigators and 10 % randomly selected respondents were revisited by supervisors for validating the data, as a quality check measure.

Gender was taken as a dichotomous variable. Age was taken as a continuous variable. Caste and sub castes (sub tribal groups) were enumerated.

Maternal education has been taken as a categorical variable. For descriptive analysis, the education was taken as ‘no education’, ‘upto 5 years of schooling (primary)’, ‘upto 8 years of schooling (middle school)’, upto 10 years of schooling (matriculation)’ and more than 10 years of schooling. For multivariate analysis, two categories were used- ‘less than 10 years of schooling’ and ‘more than 10 years of schooling’ as other studies (NFHS-3) have also shown that women with higher education levels have a strong positive impact on child survival and health. Households were ranked on wealth quintile based on assets and amenities. Asset ownership was measured with an index using Principal Component Analysis using consumer durable items and livestock in the questionnaire relevant to rural Indian setting (bedding, chair, table, cot, pressure cooker, electricity, electric fan, radio, tape recorder, black and white television, colour television, sewing machine, computer, refrigerator, mobile phone, telephone, wrist watch, clock, cycle, motor cycle, bullock cart, thresher machine, car, tractor, poultry birds, cattle and type of house- kuchcha (mud house), semi-pucca, and pucca (brick and cement construction of walls, floor and roof) (Table 1).

**Table 1:** Definition of Variables

Variable	Definition
DEPENDENT VARIABLE	
Underweight	Categorical Variable: Whether family whose children were underweight; Yes = 1 and No = 0
INDEPENDENT VARIABLES	
Sex of the child	Dummy variable = 1 if sex of the child was male, otherwise = 0 for sex of the child female
Social Status of the Family	Dummy variable = 1 if the family was ST, otherwise = 0 if family was Non-ST
Participation in any community based organization (CBO)	Dummy variable = 1 if any member of the family was participating in any CBO, otherwise = 0 if nobody from the family was participating in any CBO
Mother literacy	Dummy variable = 1 if the mother of the child possess educational qualification above Matriculation, otherwise = 0 if the mother was not educated or her education was less than matriculation.
*Asset based quintile*	
Lower 20 % ~ Quintile 1	Dummy variable = 1 if the family’s’ asset base was Lowest 20 %.
Lower 20 % ~ Quintile 2	Dummy variable = 1 if the family’s’ asset base was Lower 20 %.
Middle 20 % ~ Quintile 3	Dummy variable = 1 if the family’s’ asset base was Middle 20 %
Higher 20 % ~ Quintile 4	Dummy variable = 1 if the family’s’ asset base was Higher 20 %.
Highest 20 % ~ Quintile 5	Dummy variable = 1 if the family’s’ asset base was Highest 20 % *(Quintile 5 is a reference category)*

Households were divided into five quintiles with quintile-1 as poorest quintile and quintile-5 as least poor.

Weight was measured using similar mechanical weighing scales as are used in Anganwadi Centres, with calibration upto 500gms. For smaller babies, weight of mothers with and without the baby was taken. Two observations were made for each child average value was taken. Weighing scale instruments were adjusted for error in measurement everyday by using a one kilogram standard weight. Difference in weight measurements occurred if the child shifted position or if the weighing machine was placed on uneven surface. This was corrected by keeping the machine on a flat surface, and repeat the measurements. Date of birth was taken from Anganwadi records and triangulated with Mother and Child Protection (MCP) cards.

Data entry was done in MS Access software. Weight for Age Z-scores were calculated using Emergency Nutritional Assessment (ENA) Software. Bivariate and multivariate analysis done using SPSS 20 package for analysing the social determinants of under-nutrition.

For multivariate analysis, presence of underweight measured as -2SD or less for weight-for-age was taken as dependent variable. Several households’ demographic and socio-economic characteristics as independent variables were included in the binary logistic regression analysis. Households’ asset ownership (proxy indicator for socio-economic position) were categorised into five categories: the lowest 20 % as quintile-1, lower 20 % as quintile-2, middle 20 % as quintile-3, higher 20 % as quintile-4 and the highest 20 % as quintile-5. Quintile-5 was taken as reference group and all the other quintiles were compared with quintile-5 to see the likelihood of child being underweight. Other variables included in the analysis were gender of the studied children with male and female as categories, social status of the studied households which were divided into Scheduled Tribe (ST) and non-Scheduled Tribe categories, participation of any member of the studied households in any community based organization (CBO) and literacy level of the mother of the studied households divided into two categories- mothers with education above matriculation and mothers with no educated or less than 10 years of education. Table 1 gives the description of variables included in the logit model.

## Results

The survey covered 1070 children- 542 boys and 528 girls up to the age of 5 years. 65 % children were from tribal communities, of whom 35 % were Oraon, 16 % Munda, 11 % Khariya and 8 % from other ST groups. 35 % children were from Other Backward Classes (OBC) and general castes. In 35 % cases, mothers had no education; in 28 % cases they had studied up to fifth standard (primary) or less, in 15 % cases mothers’ education level was between standard six to eight (middle school), in 13 % cases it was between ninth to tenth standard (matriculation), and in only 7 % cases mothers had educational level of above tenth standard. Table 2 provides the descriptive statistics of the studied households and children.

**Table 2:** Respondent profile

Characteristics	Numbers	Percentages
Total participants	1070	100
Male	542	51
Female	528	49
Communities		
Oraon	324	30
Munda	172	16
Khariya	118	11
Smaller ST groups	86	8
OBC & Others	700	35
Mother’s Educational Level		
Illiterate	379	35
Upto Std 5th	303	28
Std 6–8th	164	15
Std 9–10th	144	13
Above Matriculation (Std 10th)	80	7

**Fig. 1:**
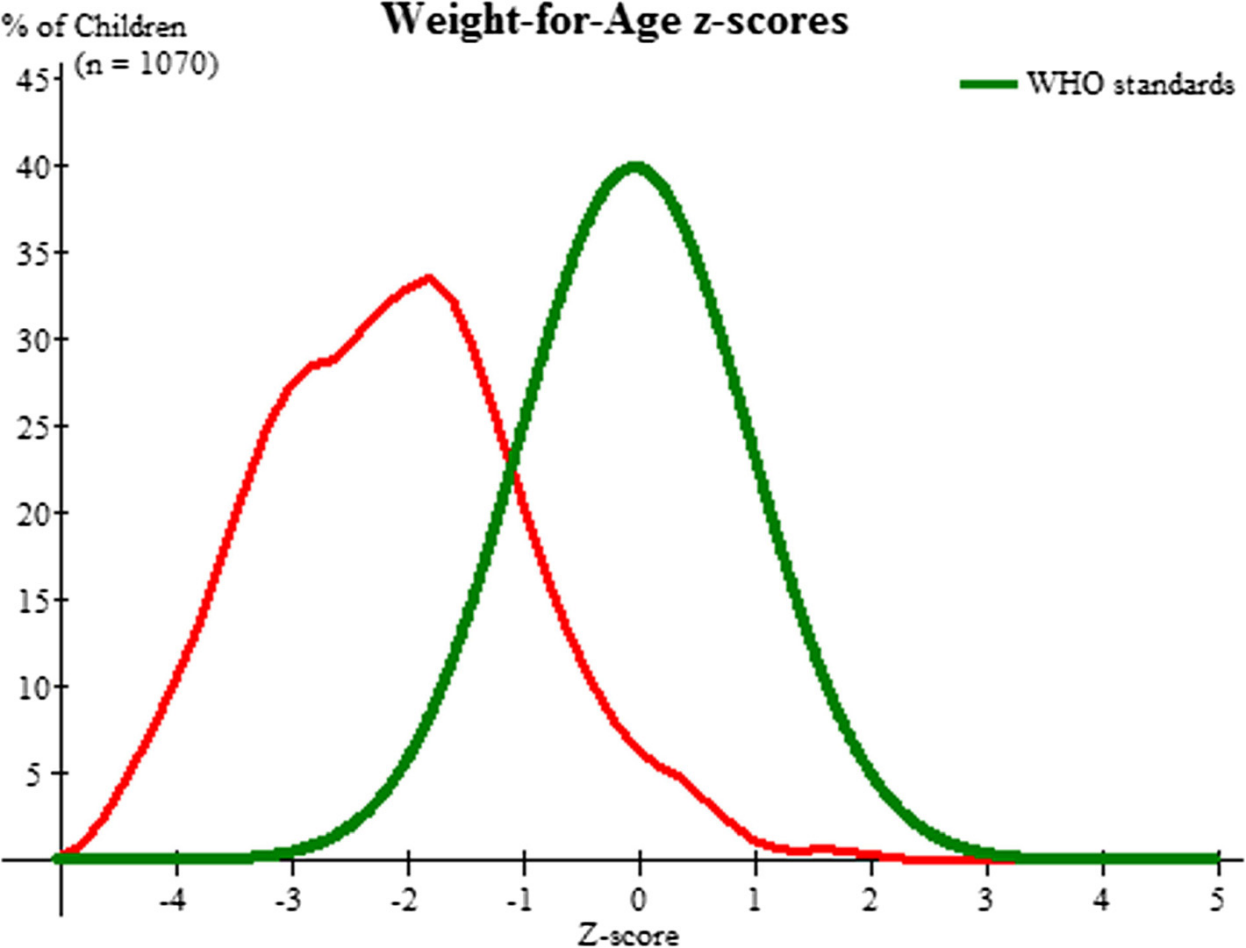
Weight for age z-score for all children

The overall prevalence of underweight was very high with 54.3 % (95 % CI 51.3–57.3) children showing less than 2SD z-score (weight -for-age), with an alarming 24.8 % (95 % CI 22.2–27.4) children showing less -3SD (severe underweight) (Fig. 1), similar to the NFHS-3 (2005–06) findings of 56.5 % children being below -2SD and 26.1 % children being below -3SD in Jharkhand.

**Fig. 2:**
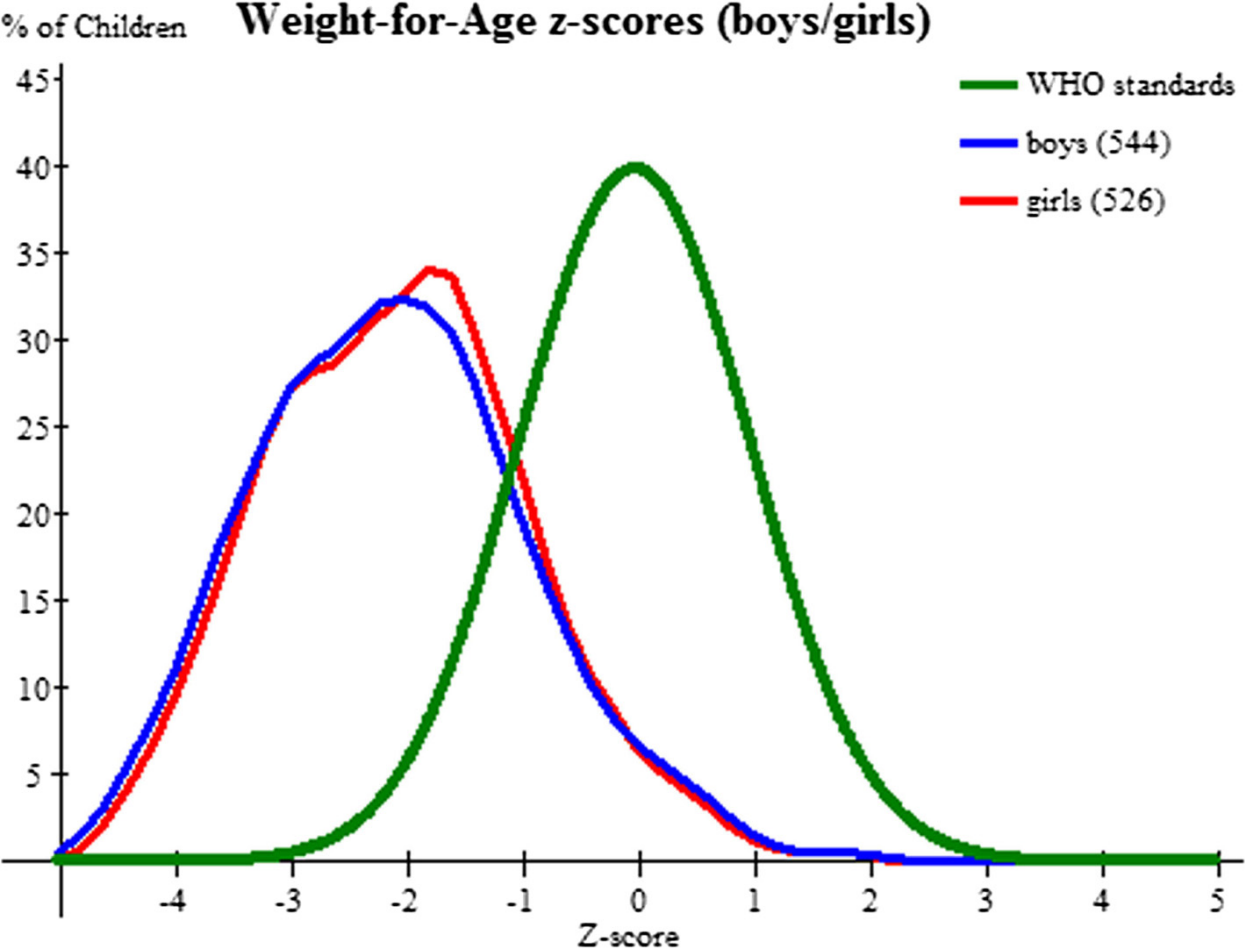
Weight for age z-score for all girls and boys

52.8 % (95 % CI 48.6 – 57.1) girls and 55.7 % (95 % CI 51.5 – 59.9) boys were underweight (<−2SD) of whom 23.5 % (95 % CI 19.9–27.1) girls and 26 % (95 % CI 22.3– 29.7) boys were severely underweight (<−3SD) (Fig. 2). However multivariate analysis did not show any significant difference between the two (Table 3).

**Table 3:** Multivariate analysis of weight-for-age

Characteristics	Coefficient (S. E.)	Exp(B)
Sex (Male = 1, Female = 0)	0.112 (0.122)	1.119
Social Status (ST = 1, Non-ST = 0)	0.002 (0.134)	1.002
CBOParticipation (Yes = 1, No = 0)	−0.031 (0.128)	.969
Motherliteracy (No Edu. Or less than Matriculation = 0, Above Matriculation = 1)	−0.168 (0.162)	0.845
*Asset Based Quintiles*		
Quintile 1	0.531 (0.210)	1.700**
Quintile 2	0.406 (0.205)	1.500**
Quintile 3	0.167 (0.198)	1.181
Quintile 4	0.480 (0.197)	1.617**
Quintile-5 was the reference group		
Constant	−0.168 (0.186)	.845
*Number of Observations*	1094	
*Significance of Hosmer and Lemeshow Chi-Square test of goodness-of-fit*	0.813	
*Significance of Omnibus test of the model*	0.060	
*Nagelkerke R-square*	0.02	
*-Loglikelihood*	1493.976	
**p < 0n05		

**Fig. 3:**
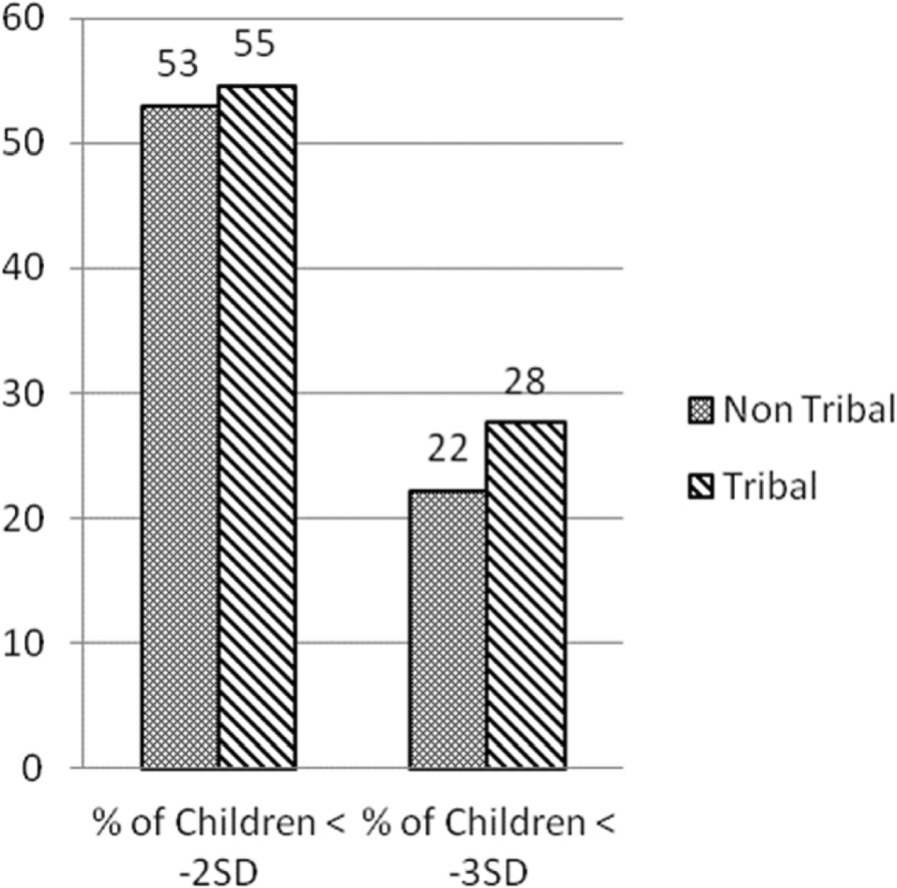
Proportion of Underweight Children in Tribal-and Non-Tribal Communities

Descriptive statistics showed that 55 % children from tribal communities and 53 % from non-tribal communities had a Z-score of less than -2SD, however, the difference was non-significant in the multivariate analysis. (Fig. 3).

22 % children from non-tribal and 28 % children from tribal communities had Z-score of less than -3SD, implying that the odds of a tribal child being severely underweight was 24 % higher than a non-tribal child (OR 1.24, 90 % CI 0.93–1.66) nearly significant at 90 % confidence level (Fig. 3). The findings closely resemble NFHS −3 data (2005–06) at national level showing 24.9 % ST children and 15.7 % OBC children were severely underweight, i.e., odds of children being severely malnourished was 78 % higher among ST group compared to odds of OBC . However, multivariate analysis comparing prevalence of underweight in Scheduled Tribe (ST) and other communities showed non-significant difference (Table 3).

**Fig. 4:**
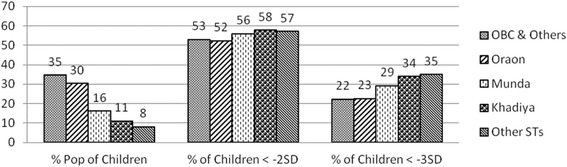
Proportion of population and nutritional status of children

However, disaggregated data for tribal communities into numerically larger (Oraon) and smaller groups showed a distinct pattern of inequality emerging. 35 % children surveyed were from OBC and other castes, 30 % were from Oraon tribal community, 16 % were Munda (ST), 11 % were Khariya (ST) and 8 % from other smaller tribal groups. Numerically smaller communities had a larger proportionate share of children with less than -2SD Z-score (Fig. 4).

Further analyses of prevalence of severe underweight, showed that about one in three children weighed less than -3SD (severe underweight) in the numerically smaller communities. The proportion was slightly less among Oraons and OBC at 23 and 22 % respectively, compared to Munda (29 %), Khadiya (34 %) and other ST groups (35 %) though still very high in all the communities (Fig. 4).

Multivariate analysis showed poverty as the single strongest predictor of under-nutrition. Likelihood of child malnutrition is significantly higher at 95 % confidence level for Quintile 1 (aOR 1.70, CI 1.13–2.57), Quintile 2 (aOR 1.50, CI 1.01–2.24) and Quintile 4 (aOR 1.62, CI 1.10–2.38) compared to Quintile 5 (Table 3). Likelihood of child under nutrition was higher in quintile 3 compared to quintile 5, however, the finding was statistically non-significant (aOR 1.18, CI 0.80–1.74).

**Fig. 5:**
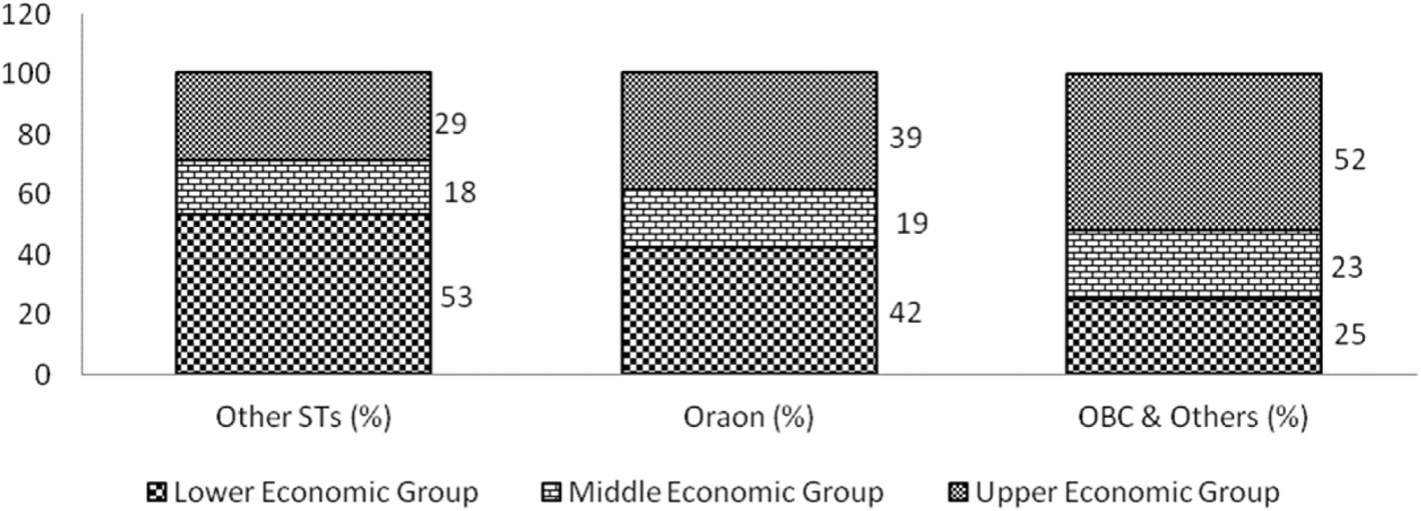
Proportion of Social Groups by Wealth Ranking

Interestingly, asset based index disaggregated for social groups showed that numerically smaller ST groups also were lower in wealth, showing multiple marginalisation. OBCs and Oraons scored better than the other communities (Munda, Khariya, other ST groups) (Fig. 5).

Maternal education is considered to be an important determinant of child’s health. Data showed that educational attainment of mothers was similar in all the communities.

**Fig. 6:**
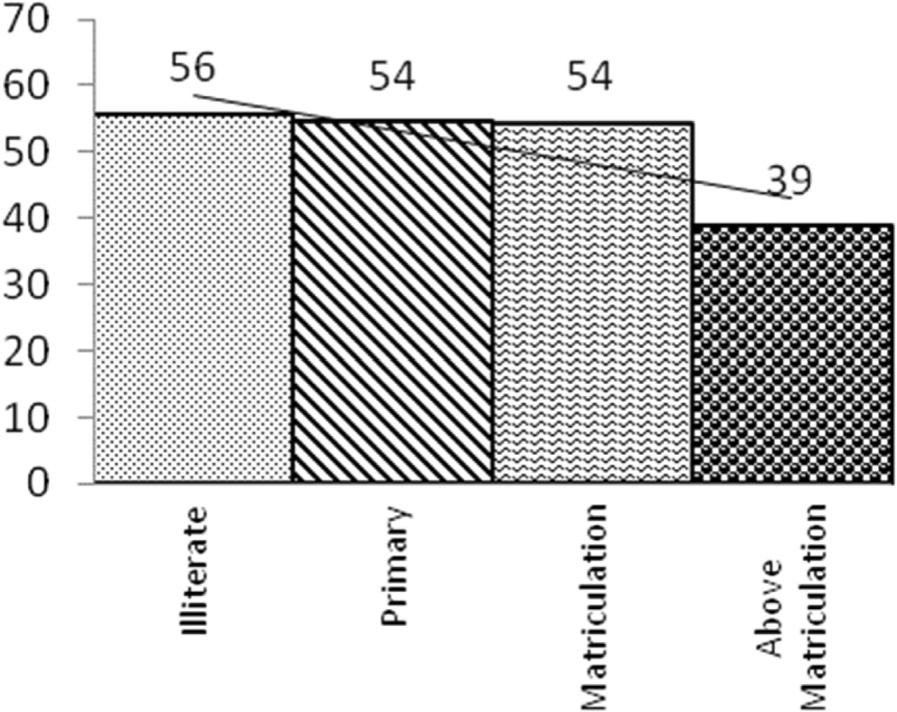
Proportion of Underweight Children (−2SD) vis-a-vis Maternal Education

Bivariate analysis showed the odds of children being underweight (<−2SD) for mothers who had upto 10 years of schooling was 43 % more than those who had more than 10 years of schooling (OR 1.43, CI 0.93–2.19) and nearly significant at 90 % confidence level (Fig. 6).

**Fig. 7:**
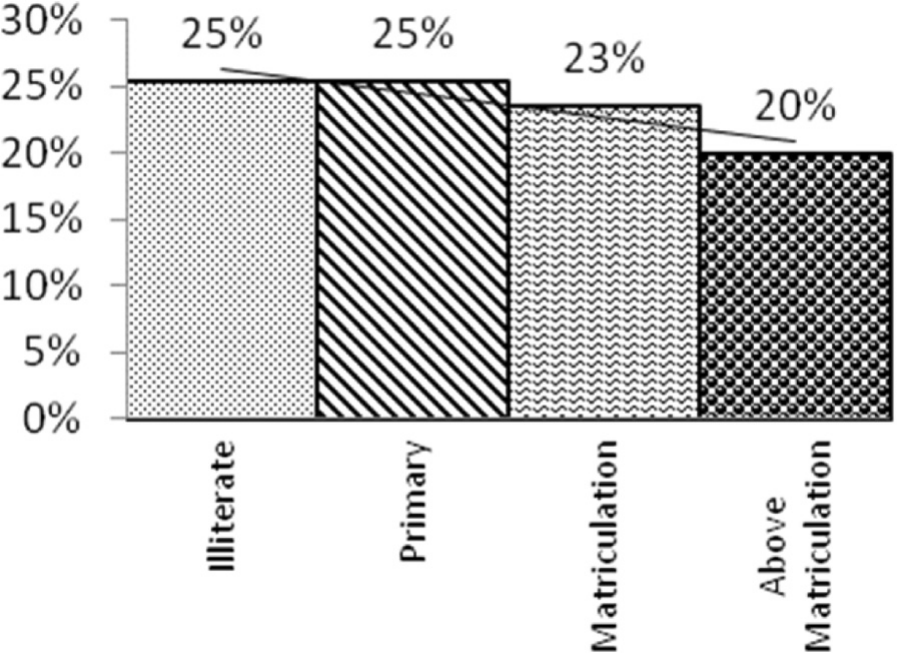
Proportion of Severe Underweight Children (−3SD) vis-a-vis Maternal Education

Odds of children being severely underweight for mothers who had less than or up to 10 years of schooling was 26 % more than those who had more than 10 years of schooling (OR 1.26, 95 % CI 0.72–2.19) (Fig. 7). Multivariate analysis also showed similar result of positive association of mother’s literacy and child underweight (aOR 0.85, 95 % CI 0.6–1.16) (Table 3).

Few studies have disaggregated tribal communities into smaller sub groups. This study shows that even ST communities are not homogenous and some sub groups are more vulnerable than the others in terms of marginalisation, who need to be effectively targeted within the larger programmes.

There were existing Women Self Help Groups in the area under study, however, there was no difference in prevalence of underweight among children whose mothers were members of such community based groups and those who were not. Interventions focussing on new-born survival had been initiated with these women’s groups during the survey period, however these activities were not specifically focussed on nutrition at that point of time.

### Limitations

The study was limited to measuring weight-for-age as a composite indicator of nutritional status of children and did not cover other indicators like stunting or wasting. The primary objective of the study was to identify groups with high prevalence of malnutrition and increase outreach of government services to such high risk groups. Weight for age, a standardised method for growth monitoring, was used to measure the magnitude of malnutrition. Government of India also uses weight-for-age for growth monitoring in its ICDS programme, and taking the same indicator helped in communicating with the government machineries, and therefore served the purpose. However, a limitation of this study is that it does not indicate whether the faltering is due to long term failure in gaining height (stunting) or short term loss of weight (wasting). Therefore for any nutrition specific intervention, it would be important to measure weight and height and if needed other micronutrient deficiencies.

Children were identified primarily from Anganwadi records and also through snowballing with health workers and women group members; it is possible that some children from excluded communities were left out from the listing. The analysis therefore is more reflective of children enrolled in the Anganwadis. Separate age bands for children were also not taken to see if there was any variation. The study could not cover particularly vulnerable tribal groups (PVTGs), considered the most disadvantaged sections, due to very small population scattered over large geographic terrain. There is a need for a separate study to understand their nutritional status.

It was a cross sectional study, and we could not take into account any seasonal variation, that is quite possible, in view of fluctuating food security situation, risk of seasonal illness and seasonal variation in occupation, especially mother’s engagement in activities like agriculture or forest produce collection, that can affect the caring time for the child. Likewise, causal relation cannot be established through this study.

## Discussion

Poverty emerged as the single most important underlying determinant of nutritional status among children in the four Blocks of Gumla district. Though this study did not specifically cover the proximate determinants, poverty plays out in form of food insecurity, poor living conditions and low access to health care, thus underpinning the importance of social security schemes and public investment for the poor households.

The impact of maternal or caregiver’s education on child health could have been mediated by household resources and socioeconomic status [17–19] and access to improved housing conditions and water and sanitation [20, 12]. Such factors as safe drinking water, sanitation and access to health care, that have a direct bearing on health need to be ensured.

More years of schooling for girls has an impact on delaying age of marriage [21–23], which subsequently, has positive health outcomes in terms of increased opportunity for a girl child for a productive adulthood, de-feminisation of poverty, reduced gender violence, along with positive impact on health outcomes like increased use of birth spacing, fewer children, reducing incidences of low birth weight or preterm babies, and reduced risk of maternal and neonatal mortality [24, 25]. Therefore there is a strong need to promote higher education among girls.

Commission on Social Determinants of Health (CSDH) has identified social participation and empowerment as a key strategy for dealing with inequities, apart from policy interventions and inter sectoral actions. There are evidences that good caring practices can mitigate the negative effects of poverty and low maternal schooling status on children’s nutritional status [26]. Women empowerment and health promotion among women using adult learning methods like Participatory Learning and Action approach (PLA) have shown evidence of improving neonatal survival in underserved and marginalised communities [27, 28] and have shown to reduce moderate depression among women, which helps in both nutritional and child development outcomes, and therefore is nutrition sensitive [29]. Such community based approaches have a potential to save lives by addressing two important social determinants- gender and poverty [30], and could therefore be integrated into the intervention package.

## Conclusion

The study shows an overall alarming situation of under-nutrition among children less than 5 years of age in Gumla, and calls for immediate intervention with universal coverage.

It further shows that tribal community is not homogenous and inequities exist even within the tribal communities. The smaller tribal communities showed signs of multiple marginalisation in terms of poverty and nutritional outcome for children. These communities need to be ‘soft targeted’ within the universal coverage.

The study highlights the importance of integrating poverty alleviation programmes with nutritional interventions, and need for promoting higher education among girls. Since the maternal educational level was similar and low for all the communities in the area, it implies that the programmes targeting promotion of higher education among SC/ST girls could be expanded for other communities as well.

There is a scope for further research on impact of social security and poverty alleviation programmes, access to water and sanitation and health care and nutrition specific community mobilisation using Participatory Learning Approach on nutritional status of children among tribal communities, disaggregating for smaller tribal groups.

## Abbreviations

ANM, auxiliary nursing midwife; ASHA, accredited social health activist; CBO, community based organisation; CSDH**,** commission on social determinants of health; ENA, emergency nutritional assessment; ICDS, integrated child development scheme; MCP, mother and child protection card; NFHS, national family and health survey; OBC, other backward classes; PCA, principle component analysis; PLA, participatory learning and action approach; PVTG, particularly vulnerable tribal groups; SC, scheduled castes; SES, socio economic segments; ST, scheduled tribe.

## Footnotes

1 Government of India, Population Census of India, 2011, Office of Registrar General and Census Commissioner, Ministry of Home Affairs, Government of India.

2 Calculated using RSBY data of targeted families. Available http://www.rsby.gov.in/Statewise.aspx?state=30 accessed on 16.8.2015.
